# Factors affecting uptake and completion of isoniazid preventive therapy among HIV-infected children at a national referral hospital, Kenya: a mixed quantitative and qualitative study

**DOI:** 10.1186/s12879-020-05011-9

**Published:** 2020-04-21

**Authors:** Serah Kajuju Ngugi, Peter Muiruri, Theresa Odero, Onesmus Gachuno

**Affiliations:** 1 Engineer County Hospital, Nyandarua, Kenya; 2 Comprehensive Care Centre, Kenyatta National Hospital, Nairobi, Kenya; 3 School of Nursing Sciences, University of Nairobi, Nairobi, Kenya; 4 Department of Obstetrics and Gynaecology, School of Medicine, University of Nairobi, Nairobi, Kenya

**Keywords:** Isoniazid, Tuberculosis, Opportunistic infections, HIV infection, Qualitative research

## Abstract

**Background:**

Tuberculosis (TB) is the most common opportunistic infection and the leading cause of death in people living with HIV (PLHIV). HIV-infected children are at a higher risk of TB infection and disease compared to those without HIV. Isoniazid preventive therapy (IPT) is an effective intervention in preventing progression of latent TB infection to active TB. The World Health Organization (WHO) currently recommends that all children aged > 12 months and adults living with HIV in whom active TB has been excluded should receive a 6-months course of IPT as part of a comprehensive package of HIV care. Despite this recommendation, the uptake of IPT among PLHIV has been suboptimal globally. This study sought to determine the factors affecting IPT uptake and completion among HIV-infected children in a large HIV care centre in Nairobi, Kenya.

**Method:**

This was a cross-sectional mixed methods study comprising of quantitative and qualitative study designs. Medical records of 225 HIV-infected children aged 1 to < 10 years, in care in the Kenyatta National Hospital Comprehensive Care Centre (KNH CCC) were retrospectively reviewed, and 8 purposively selected healthcare providers and 18 consecutively selected caregivers of children were interviewed.

**Results:**

IPT uptake among CLHIV in care in the KNH CCC was 68% (152/225) while the treatment completion rate was 82% (94/115). IPT-related health education and counselling were the main facilitators of IPT uptake and completion, while fear of adverse drug reaction, pill burden and lack of an integrated monitoring and evaluation system for IPT were the major barriers.

**Conclusion:**

The IPT uptake in this study was low and fell short of the set global target of > 90%. The completion rate was however acceptable. There is an urgent need to address the identified barriers.

## Background

Tuberculosis (TB) is the leading cause of death from infectious diseases for children of all ages globally [1]. Children with immunosuppression such as those with HIV infection are most at risk of morbidity and mortality from TB [1]. According to the WHO global TB report 2019, approximately 1.1 million children < 15 years fell ill with TB in 2018, accounting for 11% of all incident cases [2]. In the same year, about 205,000 children < 15 years (among them, 32,000 children living with HIV) died due to TB [2].

TB worsens morbidity in children living HIV (CLHIV). Unfortunately, definitive diagnosis and management of TB in these children can be quite challenging. CLHIV may have multiple and concurrent opportunistic lung infections that clinically present like TB [3]. In addition, the paucibacillary nature of TB disease and difficulty experienced in obtaining sputum specimen in children, could lead to missed or delayed diagnosis [4]. Moreover, concurrent management of both conditions can be complicated not only by high pill burden and increased risks of drug-drug interactions, but also by overlapping toxicities and immune reconstitution inflammatory syndrome (IRIS) [5].

Although antiretroviral therapy (ART) has significantly reduced the risk of life-threatening HIV- related infections such as TB, PLHIV on ART including children, still have higher TB incidence rates and a higher risk of dying from TB compared to those without HIV infection [6–10]. To reduce the burden of TB among PLHIV, the WHO recommends TB preventive therapy (TPT) as an instrumental component of HIV care [11]. TPT when used appropriately, has a synergistic effect with ART and also independently lowers the risk of TB disease among PLHIV [12, 13].

TB preventive therapy (TPT) involves the administration of one or more anti-tuberculosis drugs such as isoniazid to individuals with latent infection with *M. tuberculosis* in order to prevent progression to active TB disease [14]. TPT not only spares the individuals from the burden of TB-associated morbidity and mortality, but also reduces the economic impact of the disease on the health system as a whole [15]. For over a decade, isoniazid preventive therapy (IPT) has been a priority WHO recommended TPT for PLHIV and has been included in subsequent guidelines [11, 16, 17]. The current WHO guidelines recommend daily dose of isoniazid monotherapy for 6 months for children and adults living with HIV (including pregnant women) who are unlikely to have active TB based on a simple symptom-based screening, and children aged under 5 years who are household contacts of bacteriologically confirmed pulmonary TB cases [11].

However, despite this recommendation, TPT and IPT specifically, has remained highly underutilized [15]. In 2018, only 1.8 million PLHIV globally were reported to have been initiated on TPT of any form [18]. Although this was an improvement from just under 1 million in 2017, notably, only 16 of the 30 countries with the highest prevalence of TB/HIV in the world reported providing TPT to people newly enrolled in HIV care [18]. Coverage in these countries ranged from 10% in Indonesia to 97% in Russia [18].

There is paucity of data on IPT uptake and completion among CLHIV. The limited data available demonstrates suboptimal IPT uptake with slightly better treatment completion rates. In a cross-sectional study conducted in Ethiopia, Wasie et al. reported a low IPT uptake of 37% among CLHIV with a completion rate of 67.9% [19]. A similar study conducted in three health facilities in Nairobi, Kenya, reported a 53.2% IPT uptake with a relatively good completion rate of 88% [20]. For us to reduce the burden of TB among CLHIV and improve health outcomes in these children, every perceived barrier to TB prevention should be addressed.

Kenya is one of the 14 countries globally that are in all the three lists of the 30 high-burden countries (HBC) for TB, HIV/ TB coinfection and multidrug-resistant TB (MDR-TB) [21]. In 2017, children aged < 15 years accounted for 9.1 and 19% of all patients diagnosed with TB and TB/HIV coinfection respectively [22]. While Kenya has made great strides in reducing the overall HIV/TB co-infection rate from 45% in 2008 to 28% in 2017, these rates are still higher compared to the global estimated average of 9% in 2017 [22–24].

In line with the WHO recommendation, in 2012, Kenya adopted and rolled out the implementation of the 6-month IPT regimen among PLHIV in selected health facilities, followed thereafter in 2015 with a countrywide scale up [23]. In 2016, the country launched a 100-day national HIV testing and treatment Rapid Results Initiative (RRI) which had among its priorities a target of enrolling 90% of PLHIV on IPT by December 2016 [25]. However, by the end of that year, only 465,811(45%) of PLHIV in care had been initiated on IPT with 85,134 of them being children [25].

While suboptimal coverage of IPT among PLHIV is well documented, there is paucity of data on IPT uptake among CLHIV specifically and, limited knowledge regarding the completion rates among those initiated on IPT as well as factors that influence IPT uptake and completion in this population.

## Methods

### Aim

The aim of this study was to determine the rates of IPT uptake and completion among HIV-infected children aged 1 to < 10 years, in care in a large HIV care centre in Nairobi Kenya, and explore the factors affecting IPT uptake and completion in that population as perceived by the caregivers and healthcare providers.

IPT uptake was defined as the proportion of CLHIV in care and eligible for IPT who had been initiated on the treatment. IPT completion on the other hand was defined as the proportion of children initiated on IPT who had documented evidence of successful completion of the 6-months course.

### Setting

This study was conducted in the Kenyatta National Hospital Comprehensive Care Centre (KNH CCC), a large HIV care centre in a tertiary referral hospital in Nairobi, Kenya. Children seeking care at the centre are attended to in one wing of the building, separate from the adults. Those aged > 10 years are usually followed up in a program for adolescents and young adults dubbed ‘Triple Zero’ which empowers them to take ownership of their health and treatment and are therefore not entirely dependent on their caregivers for decision-making. Children aged < 10 years on the other hand are routinely brought to the clinic by their caregivers who play a major role in their care and treatment.

Screening for TB using a symptom-based algorithm is done at every visit. Those who are eligible for IPT are commenced on treatment and reviewed after a fortnight and thereafter monthly until they complete the treatment. Clinicians write a paper-based prescription which the caregivers present to the pharmacy for dispensing of the drugs.

### Design

This was a cross-sectional mixed quantitative and qualitative study. The quantitative component involved retrospective review of medical records of all HIV-infected children aged 1 to < 10 years receiving outpatient care at the centre for at least 3 months as of 31st March 2018. The review included medical records of all children who had been enrolled in care between 1st of January 2008 and 31st of December 2017. The qualitative component involved in-depth interviews of healthcare providers involved with the direct care of CLHIV at the centre and caregivers of children receiving care there.

### Eligibility criteria

#### Quantitative study

Children whose medical records were reviewed were aged 1 to < 10 years and had been in care for at least 3 months as at 31st March 2018. Medical records of children who were ineligible for IPT as at 31st March 2018 were excluded from the study. These included those with chronic active hepatitis and/or peripheral neuropathy and those who had been enrolled into care within the preceding 6 months and were on treatment for active TB.

#### Qualitative study

Healthcare providers who participated in the study were all working in the KNH CCC as either clinicians or pharmacy staff and were involved in the prescribing and dispensing of drugs to paediatric patients respectively. The caregivers who were included in the study had children receiving care in the KNH CCC. They all consented to participate in the study.

#### Sampling technique

For the quantitative component of the study, all 225 medical records of children who met the inclusion criteria were reviewed. For the qualitative component, purposive sampling, a non-probability sampling technique was used to select healthcare providers to be interviewed based on the aims of the study and the characteristics of the population. The aims of this study were to understand barriers and facilitators of IPT initiation and completion among CLHIV in care. Clinicians and pharmacy staff working at the KNH CCC were therefore considered key sources of this information. The clinicians sampled were those who routinely evaluate patients for IPT eligibility, counsel them, prescribe IPT and follow up patients while on treatment. On the other hand, the pharmacy staff who were sampled were those that dispense the medicine and assess adherence to treatment at each clinic visit. Caregivers were consecutively selected as they brought their children to the clinic and interviewed until a point of saturation of ideas and responses was reached. A total of 8 healthcare providers and 18 caregivers were interviewed.

#### Study tools

A data abstraction form (additional file 1) was used to abstract patients’ information from the electronic medical records (EMR) both in the clinic and the pharmacy; IQ Care (International Quality Care) and ADT (Antiretroviral Dispensing Tool) respectively. IQ Care is a robust open source EMR application system that offers a wide variety of features applicable in clinics and hospitals [26]. It is flexible and scalable and is currently in use in over 100 locations in Kenya, Uganda, Nigeria and Zimbabwe, particularly in the management of HIV/AIDS patients [26]. Even though the IQ Care EMR has a supply chain management feature for management of drugs and other consumables, in the KNH CCC, the pharmacy uses a different EMR, the ADT in which all drugs prescribed and dispensed to the patients are entered.

For qualitative data, semi-structured health care providers’ and caregivers’ interview guides (additional files 2 and 3) were used.

#### Data collection procedures

Quantitative data comprised of retrospective review of prospectively collected data of all HIV-infected children who were enrolled in care between 1st of January 2008 and 31st of December 2017 and had been in care for at least 3 months as of 31st March 2018. Using IQ Tools, a data mining functionality in the IQ Care system, a list of patient identification numbers for children to be studied was extracted along with pre-defined variables such as age, gender, date of HIV diagnosis, WHO HIV staging at enrolment, history of tuberculosis, whether or not screened for tuberculosis, whether or not the patients were prescribed for isoniazid and whether or not they completed 6 months of IPT. Individual medical records from IQ Care were compared with records in ADT in pharmacy to establish whether isoniazid was dispensed and for what duration.

Qualitative data was collected by means of in-depth interviews of health care providers involved in the prescribing (doctors and clinical officers) and dispensing of drugs (pharmacists and pharmaceutical technicians) and, caregivers of HIV-infected children in care in the KNH CCC. The interviews sought to explore the facilitators and barriers of IPT uptake and completion and were all conducted by the first author. Each interview lasted approximately 15 min and responses to questions were transcribed verbatim.

#### Data analysis strategy

Quantitative data: Abstracted data was double entered and verified. Data analysis was done using IBM SPSS Version 21.0. Descriptive statistical analysis was performed. Categorical variables were presented as percentages and continuous variables as means or medians. Uptake and completion rates of IPT were presented as proportions. A modified Poisson regression model with robust error variance was used to estimate prevalence ratio (PR) as measure of association for the relationship between the independent variables (gender, age, WHO HIV staging at enrolment, history of TB disease, latest HIV viral load) and the primary outcomes which were IPT uptake and completion. A *p*-value < 0.05 was considered statistically significant.

Qualitative data: Data from the in-depth interviews was analyzed thematically using Braun and Clarke’s six-phase framework [27]. Preliminary codes were generated to describe the content, themes identified, reviewed and defined.

## Results

The demographic and clinical characteristics of children whose medical records were reviewed are as presented in Table 1. Males were slightly more than the females and a large proportion of the patients were aged between 5 and < 10 years. All patients were on the 1st line ART regimen which was abacavir (ABC), lamivudine (3TC) and lopinavir/ritonavir (LPV/r) with most of them (73%) having been on follow up for more than 3 years.

**Table 1:** Demographic and clinical characteristics of the 225 children whose records were reviewed (*n* = 225)

Characteristic	*n* (%)
Gender	
Male	118 (52.0%)
Female	107 (48.0%)
Age categories	
Between 1- < 3 years	40 (18.0%)
3- < 5 years	40 (18.0%)
5- < 10 years	145 (64.0%)
WHO stage at enrolment	
Stage 1	88 (39.0%)
Stage 2	63 (28.0%)
Stage 3	47 (21.0%)
Stage 4	12 (5.3%)
Not documented	15 (6.7%)
On ART	
Yes	225 (100.0%)
No	0
Duration of care in KNH CCC	
< 3 Years	60 (26.7%)
> 3 years	165 (73.3%)

### IPT uptake rate

Of the 225 children eligible for IPT, 152 had been initiated on IPT, giving an IPT uptake rate of 68%. A significant association was noted between children aged between 5- < 10 years and IPT uptake, with a prevalence ratio of 1.92 (95% CI 1.09–3.37, *p* value = 0.003) as shown in Table 2.

**Table 2:** Factors associated with IPT uptake

Variable	IPT uptake		PR (95% CI)	*P* Value
	Yes (*N* = 152) *n* (%)	No (*N* = 73) *n* (%)		
Gender				
Male	85 (65.0)	33 (35.0)	1.15(0.95–1.38)	0.136
Female	67(62.0)	40 (38.0)	1	–
Age categories				
< 3 years	8 (36.0)	14 (64.0)	**1**	–
Between 3- > 5 years	14 (45.0)	17 (55.0)	1.24 (0.63–2.44)	0.539
5- < 10 years	121 (70.0)	52 (30.0)	1.92 (1.09–3.37)	**0.003**
Latest viral load				
Virally suppressed	128 (71.0)	53 (29.0)	1.06 (0.80–1.41)	0.663
Not suppressed	18 (67.0)	9 (33.0)	1	–
Not documented	6 (35.0)	11 (65.0)	–	–
History of TB treatment				
Yes	15 (60.0)	10 (40.0)	**1**	–
No	137 (69.0)	63 (31.0)	1.14 (0.82–1.59)	0.401
Baseline WHO HIV staging				
1	65 (73.9)	23 (26.1)	**1**	**–**
2	38 (60.3)	25 (39.7)	0.81 (0.64–1.03)	0.083
3	35 (74.5)	12 (25.5)	1.01 (0.82–1.24)	0.948
4	6 (50.0)	6 (50.0)	1.4 (0.29–6.8)	0.67
Not documented	8 (53.3)	7 (46.7)	–	–

### IPT completion rate

Of the 152 children initiated on IPT, 37 (25%) had initiated the treatment within the preceding 6 months and were therefore still on medication at the time of the study. The remaining 115 (75%) had received IPT more than 6 months prior, with 94 of them having documented information of successful completion of the 6 months’ course. This translated to a completion rate of 82%.

Among the 21 children who did not complete treatment, 2 were reported to have stopped taking the medication due to adverse drug reaction while for the remaining 19, no reason was provided. Fifteen (71%) of those who discontinued the treatment did so within the first 2 months. In bivariate analysis, there was a statistically significant association between IPT completion and viral suppression as indicated by the previous viral load examination as shown in Table 3.

**Table 3:** Factors associated with IPT completion

Variable	IPT completion		PR (95% CI)	*P* Value
	Yes (*N* = 94) *n* (%)	No (*N* = 21) *n* (%)		
Gender				
Male	54 (79.4)	14 (20.6)	1	
Female	42 (85.7)	7 (14.3)	1.08 (0.91–1.28)	0.396
Age categories				
< 3 years	1 (50.0)	1 (50.0)	1	–
Between 3- > 5 years	2 (50.0)	2 (50.0)	1.0 (0.18–5.46)	0.999
5- < 10 years	91 (83.5)	18 (16.5)	1.67 (0.42–6.69)	0.342
Latest viral load				
Virally suppressed	91 (84.3)	17 (15.7)	2.1 (0.72–6.18)	**0.041**
Not suppressed	2 (40.0)	3 (60.0)	1	–
Not documented	1 (50.0)	1 (50.0)	–	–
History of TB treatment				
Yes	5 (83.3)	1 (16.7)	**1**	–
No	89 (81.7)	20 (18.3)	0.98 (0.68–1.42)	0.988
Baseline WHO HIV staging				
1	45 (76.3)	14 (23.7)	**1**	**–**
2	20 (87.0)	3 (13.0)	1.1 (0.92–1.41)	0.305
3	20 (90.9)	2 (9.1)	0.8 (0.38–1.97)	0.713
4	3 (75.0)	1 (25.0)	0.98 (0.54–1.76)	0.911
Not documented	6 (85.7)	1 (14.3)	–	–

### Factors affecting IPT uptake and completion as perceived by healthcare providers and children’s caregivers

A total of 18 caregivers and 8 healthcare providers participated in the qualitative study. Characteristics of the participants are as shown in Tables 4 and 5. Most of the caregivers were females (83%, 15/18) with the largest proportion of them (87%, 13/15) being biological mothers. Almost 9 out of 10 of the caregivers reported that their children had been in care at the KNH CCC for at least 1 year. Of note is that while a vast majority (78%, 14/18) of caregivers reported that they were aware of IPT, about a third (6/18) of them reported that their child/children had not received IPT. Four of them reported that they had not been informed that their children needed to be on IPT while two reported to have declined the treatment with one citing pill burden and the other fear of side effect (peripheral neuropathy) as the reasons for declining treatment for their children. This information was consistent with their medical records which revealed that IPT had been deferred in four of the children and that caregivers and declined treatment in the other two. In the former, no reasons had been documented for deferring the treatment.

**Table 4:** Characteristics of the caregivers interviewed (*n* = 18)

Characteristic	***n*** (%)
Gender	
Male	3 (16.7)
Female	15 (83.3)
Age	
< 30 years	2 (11.1)
30 < 45 years	12 (66.7)
45 < 60 years	3 (16.7)
> 60 years	1 (5.6)
Relationship of caregiver to child	
Mother	13 (72.3)
Father	3 (16.7)
Other	2 (11.0)
Awareness of IPT	
Yes	14 (77.8)
No	4 (22.2)
History of IPT use in the child	
Yes	12 (66.7)
No	6 (33.3)
Duration of child’s care at KNH CCC	
< 1 Year	2 (11.1)
> 1 year	16 (88.9)

**Table 5:** Characteristics of the healthcare providers who were interviewed (*n* = 8)

Characteristic	***n*** (%)
Gender	
Males	2 (15.0%)
Females	6 (75.0%)
Age	
< 30 years	2 (15.0%)
30–60 years	6 (75.0%)
Professional cadre	
Clinicians: Medical doctors	4 (50.0%)
Clinical officers	1 (12.5%)
Pharmacy staff: Pharmacist	1 (12.5%)
Pharmaceutical technicians	2 (25.0%)
Years of service in the KNH CCC	
< 1 Year	1 (12.0%)
> 1 year	7 (88.0%)

Majority of the healthcare providers interviewed were females and clinicians (doctors and clinical officers). Almost all of them had been working in the KNH CCC for more than a year.

From the in-depth interviews with the healthcare providers and children’s caregivers, three key themes emerged as affecting uptake and completion of IPT among HIV-infected children in the KNH CCC, namely; IPT-related health education and counselling, drug-related factors and health information system-related factors

1. **IPT-related health education and counselling.**
  Adequate IPT-related health education and counselling were cited by both caregivers and health care providers as the two key facilitators of IPT uptake and completion.
  *“When the doctor told me that my child and I were at risk of getting TB …*. *and, that there is a drug that we can take to prevent it, we took and completed the 6 months.” –* Caregiver.

“*… .. I met a couple who had not received IPT and had declined to have their child initiated on IPT citing unpreparedness. When I took time to allay their fears and concerns about IPT, they accepted to initiate IPT and are all doing well” -*Clinician.

Insufficient caregivers’ knowledge of IPT and, inadequate counselling regarding the duration of IPT was conversely, a barrier to IPT uptake and completion respectively. One male caregiver whose child had not received IPT reported that he was not aware that his child was eligible for IPT since he was virally suppressed.

*“Though I have heard about that drug, I have never been told that he needs it …*. I *think it’s because he has no problem and his viral load was zero.” –* Caregiver.

Another caregiver whose child took IPT for only one month reported that he thought that his child had completed the whole course of treatment.

*“She completed the medicine she was given for one month … … I did not know that she needed to take for six months.”-* Caregiver.

## Drug-related factors

Two main drug-related factors were reported to influence IPT uptake; fear of potential adverse drug reactions and pill burden.

- Adverse drug reactions.

Fear of isoniazid- associated hepatotoxicity was reported by the healthcare providers as a cause for reluctance in prescribing IPT among clinicians. Their fear is reported to have resulted from previous incidences where two HIV-infected adults at the KNH CCC developed hepatotoxicity following IPT administration and subsequently succumbed to hepatic failure.

*“When we started providing IPT, we lost two adult patients due to hepatotoxicity … ..You know, these were patients who were doing well on their ART and were virally suppressed. This made clinicians to stop prescribing Isoniazid for quite some time.” –* Clinician.

“*…*. *because of those two mortalities, up until now, some clinicians are still not confident enough to prescribe IPT.” –* Clinician.

One of the caregivers whose child had not yet received IPT reported that she had declined to initiate her child on IPT because she had experienced peripheral neuropathy herself and did not want her child to go through the same.

“*…*. *you know my hands and feet felt as though they were on fire …*. *I could not walk well or hold anything. I fear that she might experience the same.”* Caregiver.

b) Pill burden.

Pill burden was reported as another reason influencing uptake and completion of IPT. This was especially so if the child was on ART regimens which are not fully fixed-dose combinations.

*“Assuming a child weighs 15 kgs and is on Abacavir, Lamivudine and lopinavir/r …*. *that means he/she gets 4 pills per day of ARVs and 2 tablets of cotrimoxazole. When you add 2 tablets of isoniazid and 1 tablets of pyridoxine, the pill burden becomes quite high … .. a caregiver of such a child may declines to have the child initiated on IPT and if they do, they may not complete the 6-months course.”* Clinician.

*“My child has problems taking even the ARVs, adding more medications now will be too much for her. Maybe later when she gets used to the ARVs.”* Caregiver*.*

2. **Health information management system-related factors**
  Health information system-related factors were cited by both the clinicians and the pharmacy staff as influencing the monitoring and evaluation of IPT and in effect the uptake and completion of IPT. Key factors identified included;

- Lack of interoperability between the clinic and pharmacy databases.
  It was noted that at the KNH CCC, the clinic and the pharmacy run two independent databases, IQ Care and ADT databases for the clinic and pharmacy respectively. The clinicians enter the patients’ information in the IQ Care database and then write paper-based prescriptions that the patients present to the pharmacists to have the drugs dispensed. The pharmacists then enter the drugs written on the prescription into the ADT database before dispensing the drugs to the patients. The healthcare providers reported that in some instances, patients’ information is lost across the care continuum.
  *“Sometimes the clinician may initiate a patient on IPT but for some reason, the patient fails to present the prescription to the pharmacist. The pharmacist has no way of knowing this and will therefore dispense only the ARVS … .. The patient’s records in the clinic will therefore show that the patient was started on IPT but there will be no records in the pharmacy.”-* Pharmacist.
- Inadequate navigation functionality on the IPT page of the web-based IQ Care EHR.
  While all clinicians had no challenges using the symptom-based TB screening algorithm, there were disparities in the ticking of the checkboxes of IPT status in the databases. This was occasioned by some missing navigation functionality on the IPT page of the IQ Care EHR. The clinicians reported that whereas patients are screened for TB at every visit, the system does not make it mandatory to update information of the patients’ IPT status. As a result of this, some patients who for instance had IPT deferred at some point, the IPT status remained ‘deferred’ even when they later become eligible for the treatment.
  *“There is no uniformity among the clinicians in the filling of the IPT page … … some fields to be filled that should be mandatory are not. As a result of this, I think we miss some patients who require to be initiated on IPT or have their IPT refilled.”-* Clinician.
  *“I think the IPT page is a bit complex …*. W*e are not able to update the information at every visit” -*Clinician*.*

**Fig. 1:**
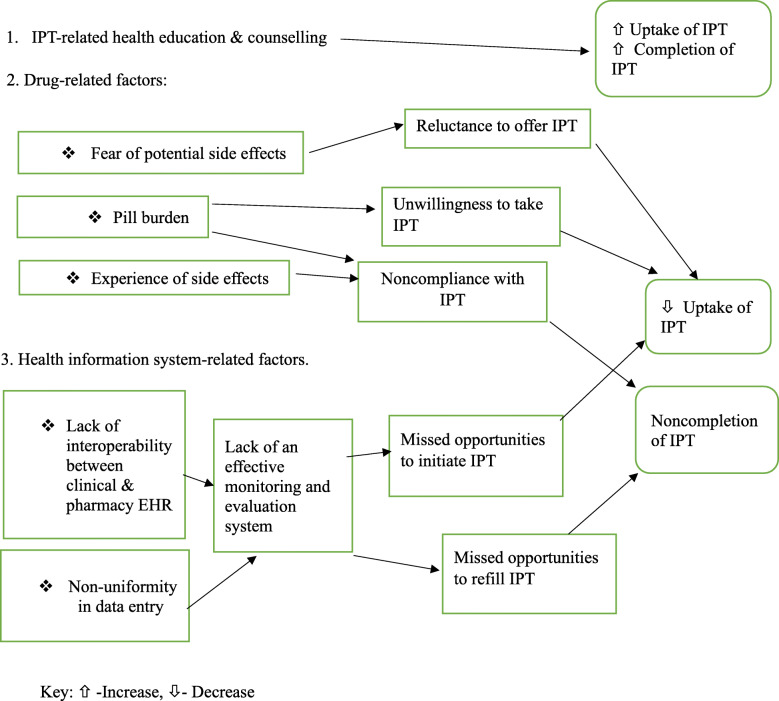
Conceptual framework of facilitators and barriers of IPT uptake and completion among HIV-infected children in care in the KNH CCC

Figure 1 summarizes the facilitators and barriers of IPT uptake and completion among HIV- infected children in the KNH CCC as deduced from this study.

## Discussion

This was a mixed quantitative and qualitative study that evaluated the uptake and completion rates of IPT and, explored factors affecting these outcomes among HIV-infected children aged 1 to < 10 years receiving care at the Kenyatta national Hospital Comprehensive Care Centre.

In this study, almost 7 in 10 (68%) of the studied children had been initiated on IPT. This uptake rate, though lower than the set global target of > 90%, was slightly higher than 53.2% reported among children aged 1 to < 15 years in three health facilities in Nairobi and also higher the national average of 45% reported in 2016 among all PLHIV in Kenya [20, 25, 28]. Similarly, a cross-sectional study conducted in Ethiopia among CLHIV aged 1 to < 15 years reported a lower IPT uptake rate of 37% [19]. The relatively higher rate documented in children in our study may be because our study did not include children aged > 10 years. These, being adolescents may have unique challenges which may be different from those of younger children who are largely dependent on their caregivers.

The IPT completion rate in this study was 82%. Though relatively good and similar to the completion rate of 88% reported by Mwangi PM et al. in Nairobi, it was lower than the 92% reported during the piloting of the IPT program in the country [20, 29]. The latter, having been a pilot study may have been more controlled compared to the routine setting where our study was carried out which reflects the real-life experiences and challenges in IPT implementation. Our study also reported a higher IPT completion rate than that reported in Ethiopia of 67.9% [19]. This higher rate may be partly explained by the fact that all patients in our study were on ART. Being on ART was associated with a higher IPT completion rate in Brazil [30]. It is hypothesized that patients who are already on ART are accustomed to taking daily medications and therefore less likely to perceive IPT as a burden [31]. In addition, they have an incentive to come to the clinic already that is independent of IPT therapy thus removing any additional travel and time burden related to acquiring IPT medication.

To gain a better understanding of factors influencing uptake and completion of IPT among children in the KNH CCC, in-depth interviews of health care providers and caregivers were conducted. Four major factors were cited; IPT-related health education and counselling, fear of pill burden, fear of adverse drug reactions and inadequate tracking of patients due to lack of integration of the clinic and pharmacy databases.

The study found that IPT-related health education and counselling were the major facilitating factors for uptake and completion of IPT. Health education is a critical process in motivating patients to adopt behaviours that are beneficial to their health [32]. When patients and caregivers of children understand the role of IPT in prevention of TB, they easily make informed decisions and accept the intervention when offered. Understanding the duration of the treatment also helps the patients to be adherent. A study in Thailand highlighted misunderstanding regarding duration of IPT as one of the reasons for defaulting treatment [33].

Some of the barriers to IPT uptake and completion that were reported by the health care providers and caregivers included, potential adverse drug reaction, pill burden and, a number perceived the 6-months course to be too long. The risk of serious isoniazid-associated toxicities can be reduced by adequate patient/caregiver health education and close monitoring [34]. Patients should be warned about symptoms of isoniazid toxicity and told to stop taking the drug and return for evaluation if these occurs. PLHIV who are on IPT should have regular follow-up clinics to monitor for symptoms of isoniazid toxicity and, also for TB screening.

In this study, fear of pill burden was reported by caregivers as the reason for declining IPT for their children. Pill burden has also been reported to contribute to poor adherence [35]. Healthcare providers reported that the fear of pill burden was more likely if the patients was already on multiple other individual drugs such as the antiretroviral drugs and cotrimoxazole. Fixed dose combination (FDC) of drug can improve adherence by reducing pill burden. FDC of isoniazid, pyridoxine and cotrimoxazole has been proposed as preventive therapy to reduce mortality and hospitalization in adults living with HIV [36]. However, the use of FDC of these drugs in the paediatric population is yet to be approved.

Several healthcare providers and caregivers perceived the currently recommended 6-months course of IPT to be too long. In 2018, the WHO published updated guidelines that recommend TB preventive therapy options which may help overcome some of the experienced or perceived challenges of IPT such as the long course of treatment [11]. In the new guideline, WHO recommends short-course, rifamycin-based TPT regimens as alternatives to the 6 months isoniazid monotherapy for both adults and children. These regimens are high-dose isoniazid and rifapentine given weekly for 3 months (referred to as 3HP) recommended for adults and children > 2 years of age, and 3 months of daily isoniazid and rifampicin recommended for children and adolescents < 15 years of age [11, 37]. These short course regimens once available will most likely improve uptake and adherence. However, these regimens containing rifampicin and rifapentine should be prescribed with caution to PLHIV who are on ART because of potential drug-drug interactions. The WHO advises that these regimens should not be administered to people receiving protease inhibitors or nevirapine [11].

Lack of an effective IPT monitoring and evaluation system was cited by healthcare providers as partly contributing to low initiation and completion of IPT. While all clinicians reported that they had no challenges using the symptom-based algorithm to screen for TB, most of them reported that the IPT page on the system was complex and as a result there were disparities and non-uniformity in the ticking of the checkboxes on the page. Lack of interoperability between the clinic and pharmacy electronic health systems, use of paper-based prescriptions were reported to result in loss of some patients’ information making it difficult to effectively track patients from the point of IPT initiation to completion. Improvement of the IPT page functionality in the electronic systems both in the clinic and pharmacy and synchronization of the two systems will help in the monitoring of the patients on monthly basis while on IPT.

## Conclusion

The IPT uptake among CLHIV in this study fell short of the set global target. The IPT completion rate on the other hand, though acceptable can be improved. IPT-related health education and counselling were the main facilitators of IPT uptake and completion, while fear of adverse drug reaction, fear of pill burden and lack of an effective monitoring and evaluation system for IPT were the major barriers.

## Recommendations

Based on the findings of this study, we recommend intensification of IPT-related health education and counselling, adoption of short-course TB preventive treatment regimens and establishment of an effective IPT monitoring and evaluation system in the HIV care centre.

## Supplementary information

**Additional file 1.** Form. Data abstraction form

**Additional file 2.** Interview guide. Caregivers’ interview guide

**Additional file 3.** Interview guide. Healthcare providers’ interview guide

## Data Availability

The data that support the findings of this study are available from the Kenyatta National Hospital administration, but restrictions apply to the availability of these data, which were used under license for the current study, and so are not publicly available. Data are however available from the authors upon reasonable request and with permission of the Kenyatta National Hospital administration.

## Abbreviations

ADT: Antiretroviral administration tool
ART: Antiretroviral therapy
CCC: Comprehensive Care Centre
EMR: Electronic medical records
FDC: Fixed-dose combination
HIV: Human immunodeficiency virus
IC: infection control
ICF: intensified case finding
IPT: Isoniazid preventive therapy
IQ: International quality
KNH: Kenyatta National Hospital
MDR: Multidrug resistant
PLHIV: People living with Human immunodeficiency virus
RRI: Rapid results initiative
SPSS: Statistical Package for the Social Sciences
TB: Tuberculosis
WHO: World Health Organization
