# Supplementary material for: Factors affecting uptake and completion of isoniazid preventive therapy among HIV-infected children at a national referral hospital, Kenya: a mixed quantitative and qualitative study
Source: BMC Infect Dis. 2020 Apr 21;20:294. doi: 10.1186/s12879-020-05011-9 (PMC7362518; doi:10.1186/s12879-020-05011-9)
Supplement: Supplementary file 1 — Additional file 1. Form. Data abstraction form [file 12879_2020_5011_MOESM1_ESM.pdf]

APPENDIX I DATA ABSTRACTION FORM **Study Number:_____________**

ISONIAZID PREVENTIVE THERAPY UPTAKE AND COMPLETION AMONG HIV- INFECTED CHILDREN AGED 1 TO <10 YEARS AT THE COMPREHENSIVE CARE CENTRE, KENYATTA NATIONAL HOSPITAL.

1. What is the gender of the child? 1= Male 2=Female
2. What's the child's current age? _____________
3. What was the age at enrolment? _____________
4. Duration of follow up___________________
5. What was the WHO HIV stage at enrolment ___________
6. What was the initial viral load? ___________
7. What is the latest viral load______________
8. Is the child on ART? 1= Yes 2=No

   8a. If yes, what regimen is the child on? ___________________

   8b. If no, why? ____
9. Has the child had TB? 1=Yes 2= No

   9a. If yes, 1= completed treatment 2= currently on treatment
10. Was the child initiated on IPT? 1= Yes 2=No

    10a. If yes, 1 = within the last 6 months

    2= more than 6 months ago.
11. If initiated on IPT > 6months ago, is there documented evidence of completion of the 6 months' course?

    1=Yes 2=No

    11a. If did not complete the 6 months course, why?

1= Adverse drug reaction, specify__________________

2= Developed TB

3= Others, specify ______________________________

____

12. If wasn't initiated on IPT, Why?

1= contraindication, specify____

2= parent/ guardian declined

3= Not documented

4= Others, specify ____

____

13. Child's IPT status as indicated on the database

1= Started 2= Deferred

3= Declined 3= completed

4= Not documented
