# Supplementary material for: Factors affecting uptake and completion of isoniazid preventive therapy among HIV-infected children at a national referral hospital, Kenya: a mixed quantitative and qualitative study
Source: BMC Infect Dis. 2020 Apr 21;20:294. doi: 10.1186/s12879-020-05011-9 (PMC7362518; doi:10.1186/s12879-020-05011-9)
Supplement: Supplementary file 2 — Additional file 2. Interview guide. Caregivers’ interview guide [file 12879_2020_5011_MOESM2_ESM.pdf]

APPENDIX VI: CAREGIVERS’ INTERVIEW GUIDE – ENGLISH

Introduction

Purpose of interview:

TB is the commonest infection in people living with HIV, particularly children because of their low immunity. Currently, it is recommended that all children more than 1 year of age should be put on a certain medicine called Isoniazid for 6 months to prevent them from getting TB. I am interested in knowing whether your child has received this medicine and what your views are concerning it.

Gender 1=male 2=female

Interview begins

- What is the relation between you and the child?
- What is you age category?
  - < 30 years
  - 30<45 years
  - 45-60 years
  - >60 years
- When was your child enrolled to the KNH CCC?
- Is your child on ART?
- Have you heard of IPT or a drug called Isoniazid?
- Tell me what you know about isoniazid or IPT?
- Has your child been given isoniazid/ IPT?
  -If yes, did they complete the 6 months of treatment? If did not complete, why?
  -If not yet given, -why?
- In your opinion, why would a caregiver decline to have their child initiated on IPT/isoniazid?
- In your view, why would a child initiated on IPT not complete the 6-months course of treatment?
- Do you have any concerns or queries regarding IPT/isoniazid?

  Thank the participant for his/her time.
