# Supplementary material for: Factors affecting uptake and completion of isoniazid preventive therapy among HIV-infected children at a national referral hospital, Kenya: a mixed quantitative and qualitative study
Source: BMC Infect Dis. 2020 Apr 21;20:294. doi: 10.1186/s12879-020-05011-9 (PMC7362518; doi:10.1186/s12879-020-05011-9)
Supplement: Supplementary file 3 — Additional file 3. Interview guide. Healthcare providers’ interview guide [file 12879_2020_5011_MOESM3_ESM.pdf]

APPENDIX III <u>HEALTHCARE PROVIDERS' INTERVIEW GUIDE</u>

FACTORS AFFECTING UPTAKE AND COMPLETION OF ISONIAZID PREVENTIVE THERAPY AMONG HIV- INFECTED CHILDREN IN THE COMPREHENSIVE CARE CENTRE, KENYATTA NATIONAL HOSPITAL.

Introduction

Purpose of interview:

We are aware that TB is the commonest opportunistic infection in people living with HIV. Currently, the World Health Organization recommends that all children more than 1 year of age should be started on Isoniazid Preventive Therapy (IPT) for 6 months after ruling out active TB. I am interested in knowing your views about IPT and some of the challenges you have experienced in regard to provision of IPT to children on follow up in this centre.

Gender: 1= male 2= female

Interview begins

- What's your professional category

  1= clinician, specify ________________

  2=pharmacy staff, specify__________________

- What's your age category? 1= < 30years

  2= between 30 and 60 years
- How long have you been working as a clinician, pharmacist?
- How long have you been working in KNH CCC?
- Approximately what proportion of patients aged 1-10 years in this clinic have been started on IPT?
- What are your views concerning IPT? Its benefits, effectiveness, safety?
- How easy is it to use the symptom- based algorithm to rule out active TB in children?
- What are the major facilitators of IPT uptake?
- In your opinion, what are the biggest challenges in the provision of IPT?
- What do you think would make a clinician reluctant to prescribe IPT to children?

- Have you come across older children who have declined to take isoniazid or parents who have declined to give isoniazid to their child/children? What reasons do they give for declining?
- Have you come across patients who started INH but discontinued? What were the reasons for discontinuing?
- Have you encountered patients who have had adverse drug reaction after taking INH? What adverse events? Did that change you views concerning IPT?
- Have you had isoniazid stock outs in KNH CCC over the last three years?
- Do you have any other concerns about the use of INH in preventing TB in HIV-infected children?

Thank the participant for his/her time.
